# Tracking excess deaths associated with the COVID-19 epidemic as an epidemiological surveillance strategy-preliminary results of the evaluation of six Brazilian capitals

**DOI:** 10.1590/0037-8682-0558-2020

**Published:** 2020-11-06

**Authors:** André Ricardo Ribas Freitas, Nicole Montenegro de Medeiros, Livia Carla Vinhal Frutuoso, Otto Albuquerque Beckedorff, Lucas Mariscal Alves de Martin, Marcela Montenegro de Medeiros Coelho, Giovanna Gimenez Souza de Freitas, Daniele Rocha Queiróz Lemos, Luciano Pamplona de Góes Cavalcanti

**Affiliations:** 1 Faculdade de Medicina São Leopoldo Mandic de Campinas, Campinas, SP, Brasil.; 2 Faculdade de Medicina São Leopoldo Mandic de Campinas, Programa de Pós-Graduação em Saúde Coletiva, Campinas, SP, Brasil.; 3 Ministério da Saúde, Brasília, DF, Brasil.; 4 Universidade Federal de Uberlândia, Faculdade de Medicina Veterinária, Uberlândia, MG, Brasil.; 5 Faculdade de Medicina de Marília, Marília, SP, Brasil.; 6 Centro Universitário Christus, Faculdade de Medicina, Fortaleza, CE, Brasil.; 7 Universidade Federal do Ceará, Programa de Pós-graduação em Saúde Coletiva, Fortaleza, CE, Brasil.

**Keywords:** Excess mortality, COVID-19, Pandemic, Respiratory vírus, Epidemiological surveillance, Intelligence tool

## Abstract

**INTRODUCTION:**

In March 2020, the World Health Organization declared the coronavirus disease (COVID-19) outbreak a pandemic. In Brazil, 110 thousand cases and 5,901 deaths were confirmed by the end of April 2020. The scarcity of laboratory resources, the overload on the service network, and the broad clinical spectrum of the disease make it difficult to document all the deaths due to COVID-19. The aim of this study was to assess the mortality rate in Brazilian capitals with a high incidence of COVID-19.

**METHODS:**

We assessed the weekly mortality between epidemiological week 1 and 16 in 2020 and the corresponding period in 2019. We estimated the expected mortality at 95% confidence interval by projecting the mortality in 2019 to the population in 2020, using data from the National Association of Civil Registrars (ARPEN-Brasil).

**RESULTS:**

In the five capitals with the highest incidence of COVID-19, we identified excess deaths during the pandemic. The age group above 60 years was severely affected, while 31% of the excess deaths occurred in the age group of 20-59 years. There was a strong correlation (r = 0.94) between excess deaths and the number of deaths confirmed by epidemiological monitoring. The epidemiological surveillance captured only 52% of all mortality associated with the COVID-19 pandemic in the cities examined.

**CONCLUSIONS:**

Considering the simplicity of the method and its low cost, we believe that the assessment of excess mortality associated with the COVID-19 pandemic should be used as a complementary tool for regular epidemiological surveillance.

## INTRODUCTION

In December 2019, an increase in the number of hospitalizations for pneumonia of unknown etiology was observed in Wuhan, Hubei, China. Initially, the disease was attributed to exposure to the disease-causing virus at a seafood and wild animal market, but human-to-human transmission was subsequently confirmed. On January 7, 2020, a new coronavirus was isolated and identified as the cause of the pneumonia outbreak. On January 13, the first case in Thailand was confirmed, and on January 29, the first case in the Republic of Korea, two cases in Beijing, and one case in Guangdong were confirmed^1^. On February 11, the World Health Organization (WHO) named the disease as novel coronavirus disease (COVID-19). The main symptoms of the disease are fever, cough, myalgia, and dyspnea in the acute phase. On the same day, the International Committee on Taxonomy of Viruses (ICTV) named this new coronavirus as severe acute respiratory syndrome coronavirus 2 (SARS-CoV-2). A WHO-China Joint Mission commissioned from February 16 to 24 reported that the estimated basic reproduction number (R0) of the coronavirus was between 2 and 2.5^2^. Even before the WHO recognized COVID-19 as a pandemic (March 11), the virus had already spread to many countries. By the end of April, more than 3.6 million cases and 251.5 thousand deaths had been registered in the five continents^2^. In Brazil, the first case of COVID-19 was confirmed on February 26, 2020. After 15 days, sustained transmission was reported, and on March 17, the first death was confirmed. By the end of April 2020, more than 110 thousand cases and 5,901 deaths had been confirmed, with a mortality rate of 20 deaths per million inhabitants, with Brazil being among the top 10 countries with the highest number of deaths and collapse of the healthcare system^3^.

Despite the high number of confirmed cases and deaths in Brazil and efforts to diagnose suspected patients, it is believed that the testing efficacy is far from adequate. The COVID-19 testing laboratory network was established gradually; it was centralized first in three units of the National Influenza Center (NIC) and later included private laboratories and the state public health laboratory network^4^. The WHO’s recommendation for mass testing of COVID-19 on March 16 resulted in a global shortage of test kits. Coupled with the increase in the number of cases, the testing capacity in both public and private health networks in Brazil was compromised^4^. This could have led to an underestimation of the magnitude of the disease burden in the country, which is probably being faced by many other countries too. 

The broad clinical spectrum of the disease, which can delay the clinical diagnosis, coupled with the scarcity of laboratory resources and the overload on the healthcare network, might not make it difficult for the epidemiological surveillance systems to detect all the cases and deaths. A reliable assessment of the burden of COVID-19 is essential so that health managers and governments can plan the scale and duration of non-pharmacological interventions required to control the disease. The objective of this study was to analyze the excess number of deaths and the high mortality rate of the current SARS-CoV-2 pandemic in the capitals with the highest number of cases recorded by the routine surveillance systems in Brazil.

## METHODS

This study was based on the official public data that we used to analyze the time series of cases and deaths due to COVID-19 and estimate the excess mortality from COVID-19 in the first few months of the pandemic in six Brazilian capitals.

### Study locations

**FIGURE 1:**
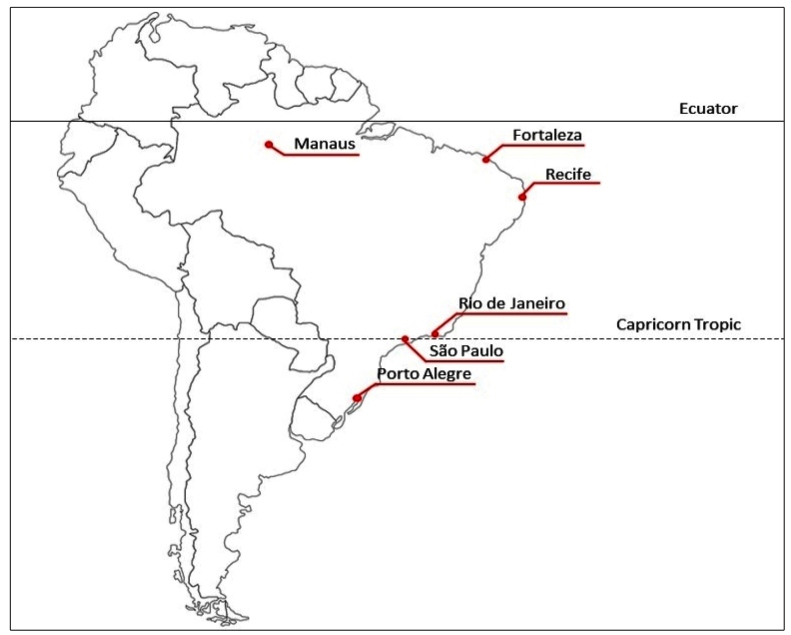
Location of the six Brazilian capitals studied.

For the present study, the five Brazilian capitals with the highest absolute numbers of confirmed cases of COVID-19 were selected: Fortaleza, São Paulo, Rio de Janeiro, Manaus, and Recife (Figure 1). To validate the proposed method of estimating excess mortality due to COVID-19, the capital state of Porto Alegre, which showed the lowest rates of COVID-19 cases and seroprevalence during a recently conducted serological survey, was also studied^5^.

### Data sources

Mortality data were collected from the website of the National Association of Civil Registrars (Associação Nacional dos Registradores de Pessoas Naturais, ARPEN-Brasil, acronym in Portuguese)^6^, which contains information on the deaths registered in notary offices across the country during 2019 and 2020. In Brazil, notary offices are given a period of 14 days to enter the data from the death certificates signed by the attending physician in the National Civil Registry Information Center (CNIRC, acronym in Portuguese). Deaths due to COVID-19, pneumonia, severe acute respiratory syndrome, septicemia, undetermined causes, and other causes were included in the analysis^7^. Deaths from external causes were excluded. Under Brazilian law, the cause of death on the death certificate must be preferably mentioned by the attending physician, and there is no obligation for laboratory confirmation. The deaths were grouped as respiratory causes (COVID-19, pneumonia, and severe acute respiratory syndrome) and all causes (respiratory causes, septicemia, other deaths, and undetermined causes).

Data of confirmed cases and deaths due to COVID-19 were entered in the repository of the Brazil project, which extracts the data from official sources following a pre-established algorithm^8^. Deaths are classified according to the date of confirmation of the disease in the Notification System for Notifiable Diseases (SINAN, acronym in Portuguese), and the data are open to the public^9^. 

To obtain the COVID-19-related mortality data from SINAN, we searched for the death dates directly in the databases and reports of the State and Municipal Health Secretariats^10^ ^-^ ^12^. It was not possible to obtain data by date of onset of symptoms for the municipalities of Rio de Janeiro and Manaus. All data were extracted on the same day (May 2, 2020).

Demographic data were collected from the Brazilian Institute of Geography and Statistics (IBGE, acronym in Portuguese) website, which estimates the population of Brazilian capitals by age group on a quarterly basis, through the Continuous National Household Sample Survey (PNAD, acronym in Portuguese)^13^. The estimated population for the first quarter of 2020 was not yet available at the time of the study, so for the first quarter of 2019, the estimated population in the last quarter of 2018 was used, and for 2020, the estimated population in the last quarter of 2019 was used.

### Data analysis

We assessed the weekly mortality from all causes during 2020 up to epidemiological week (EW) 16 and compared it with that for the corresponding period in the previous year. We calculated the expected mortality in 2020 at 95% confidence interval, by projecting the 2019 mortality rate to the population in 2020.

### Definition of the period with excess mortality

The period with excess mortality was defined as the period between the first two consecutive weeks with the number of deaths above the upper limit of 95% confidence interval and first two consecutive weeks with deaths below this limit^14^.

Since the mortality data can be entered up to 14 days after death, we evaluated the correlation between the cases officially confirmed by the Health Departments and the excess of deaths until EW 16 and constructed a linear regression curve.

The mortality rate by age group was calculated for the period with excess mortality. The expected number of deaths by age group was calculated by projecting the 2019 death rate by age group to the 2020 population. Excess mortality by age group was calculated as the number of deaths observed minus the number of expected deaths. The ratio of mortality rates was calculated by dividing the mortality rate by age group during the period with excess mortality in 2020 by the mortality rate observed in 2019 during the same period. We constructed a linear regression curve based on the number of deaths confirmed by COVID-19 in each city and the estimated number of excess deaths.

Statistical analyses were performed using the STATA 9.2 program and the results are presented in graphs and tables built in the Microsoft® Excel® program.

## RESULTS

Table 1 shows the population and the epidemiological data of the cities examined. We also present the number of excess deaths from all causes during the period with excess mortality. The rate of mortality per 100,000 inhabitants was the lowest at 30.6 in Porto Alegre and the highest at 229.2 in Recife. 

**TABLE 1:** Population, COVID-19 cases, deaths due to COVID-19, and excess mortality in six Brazilian capitals (data collected on May 2, 2020).

	Population (x1,000)	COVID-19 Confirmed cases¹	COVID-19 Confirmed deaths¹	Excess deaths	Excess deaths/ Confirmed	IR²
Fortaleza	2,667	6,082	413	193	47%	228.0
Manaus	2,197	3,491	357	1355	380%	158.9
Recife	1,649	3,780	250	444	178%	229.2
Rio de Janeiro	6,729	6,189	574	1350	234%	92.0
São Paulo	12,278	19,085	1607	2002	125%	155.4
Porto Alegre	1,485	455	15	-		30.6

¹Source (SINAN, official health surveillance system, on May 2, 2020). ²IR: Incidence Rate (cases/100,000 population).

**FIGURE 2:**
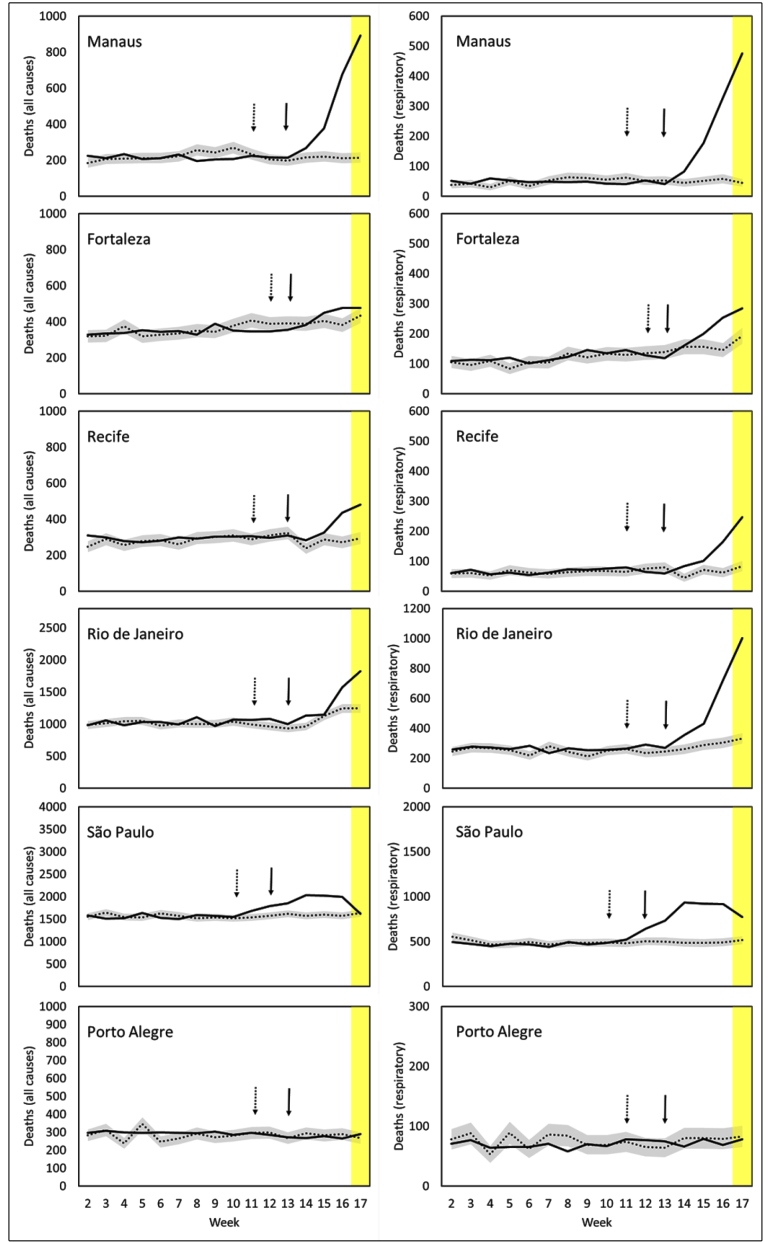
Weekly mortality observed in 2020 (black line); expected (dotted) 95% confidence interval (gray area) for respiratory causes (right) and for all causes (left) by city. The dotted arrow indicates the date of confirmation of the first case and the black arrow indicates the date of the first confirmed death in the city. The yellow area represents the period for which the mortality data are still incomplete.

**FIGURE 3:**
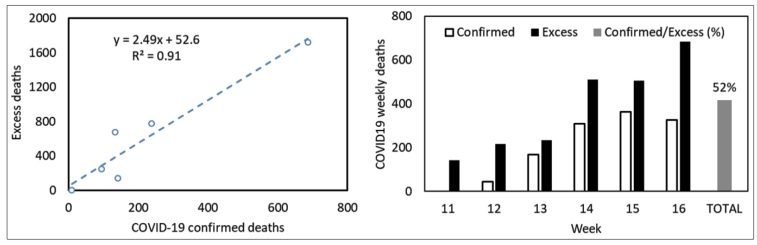
On the left **(A)**, correlation between mortality officially confirmed by COVID-19 in the cities examined and the estimated excess mortality; the equation suggests that for each notified patient, there were 2.5 excess deaths by week 16. On the right **(B)**, officially confirmed deaths by COVID-19 and excess deaths per epidemiological week. Data for the cities of São Paulo, Recife, and Fortaleza are shown according to the date of death.

Excess mortality was observed in the five cities with the highest incidence of COVID-19, but not in Porto Alegre, where there was a low incidence of COVID-19 (Figure 2). In the cities of São Paulo and Rio de Janeiro, there was an increase in all-cause mortality above the 95% confidence interval from EW 11, as compared to the mortality in the same period in the previous year. In these cities, the first cases of COVID-19 were confirmed by laboratory diagnosis in EWs 12 and 13. These findings suggest that cases and deaths due to COVID-19 may have been underestimated by the epidemiological surveillance system. In the cities of Recife, Fortaleza, and Manaus, excess deaths were observed from EW 14. In Manaus, the mortality risk in EW 17 from respiratory disease (as mentioned on the death certificate) was 11 times higher than that in the same period in the last year. The mortality risk from all causes of death together showed a fourfold increase. Assuming that EW 17 probably did not include all deaths, these numbers are expected to be higher. Figure 3-A shows the strong Pearson correlation (r = 0.95) between the number of confirmed deaths in SINAN and the excess of deaths up to EW 16 in the six cities examined. The regression slope suggests that the number of excess deaths was 2.5 times higher than the official number of deaths reported in week 16 (R² = 0.91; p < 0.0001). 

Figure 3-B was constructed using data from municipalities where the mortality data for COVID-19 based on the date of death were available (Sao Paulo, Fortaleza, and Recife). The estimated mortality from COVID-19 accounted for 52% of the excess all-cause mortality.

Table 2 shows the mortality by age group for all causes. The most strongly affected age group was above 60 years, with 3,801 excess deaths from all causes. Excess deaths were also found in the population aged between 20 and 59 years, accounting for 31% of the total excess mortality from all causes (1,679 deaths).

**TABLE 2:** Excess mortality due to all causes at the beginning of the COVID-19 pandemic in the five Brazilian capitals with the highest incidence of the disease (data referring to week 17; data collected on May 2, 2020).

	Age groups	Mort rate 2019	Mort rate 2020	Rate ratio 2020/ 2019	Confidence interval (95%)	Deaths 2019	Deaths 2020	Excess deaths	p
Fortaleza	<20 years	12,4	11,3	0.92	(0.66-1.27)	82	77	-5	0.5810
	20-59 years	16,4	21,1	1.29	(1.09-1.52)	260	328	68	<0.005
	>60 years	214,9	228,4	1.06	(0.97-1.17)	868	998	130	0.1884
	**Total**							**193**	
Manaus	<20 years	18,4	15,7	0.85	(0.65-1.11)	123	111	-12	0.2270
	20-59 years	18,5	45,2	2.44	(2.10-2.86)	233	578	345	<0.0001
	>60 years	218,8	718,4	3.28	(2.97-3.64)	501	1523	1022	<0.0001
	**Total**							**1355**	
Recife	<20 years	16,0	16,0	1.00	(0.70-1.43)	70	62	-8	0.9886
	20-59 years	24,0	33,5	1.40	(1.17-1.66)	224	321	97	<0.0001
	>60 years	291,5	378,1	1.30	(1.18-1.42)	787	1142	355	<0.0001
	**Total**							**444**	
Rio de Janeiro	<20 years	16,9	15,4	0.91	(0.76-1.10)	250	218	-32	0.3333
	20-59 years	35,7	48,6	1.36	(1.27-1.46)	1362	1842	480	<0.0001
	>60 years	410,8	437	1.06	(1.03-1.10)	5776	6678	902	<0.001
	**Total**							**1350**	
São Paulo	<20 years	17,6	15,6	0,89	(0.78-1.01)	537	458	-79	0.0562
	20-59 years	32,6	41,8	1,28	(1.21-1.35)	2271	2960	689	<0.0001
	>60 years	368,4	418,2	1,14	(1.10-1.17)	8043	9435	1392	<0.0001
	**Total**							**2002**	

## DISCUSSION

We observed an excess of 5,344 deaths from all causes during the first weeks of the epidemic in the five Brazilian cities severely affected by COVID-19. This increase was pronounced in cities with a high incidence of COVID-19; 31% of the excess of deaths occurred in the population aged 20 to 59 years. These findings are in line with what has been observed in clinical studies and reported in the bulletins of Brazilian epidemiological surveillance. There were no excess deaths in the population below 20 years nor an increase in overall mortality in the city of Porto Alegre, where the incidence of COVID-19 at the time of analysis was low. These data reinforce the consistency of the findings.

The number of deaths that occurred until the EW 16, as confirmed by the official epidemiological surveillance system, corresponded to 52% of the excess deaths that occurred during the study period. Considering that the data were collected 14 days after the end of EW 16, we believe that investigations of almost all deaths would have been completed. Therefore, we can assume that the epidemiological surveillance system captured only 52% of the total mortality burden of this disease. This is not surprising since other viruses such as influenza and chikungunya also have a mortality burden that is often higher than that reported from etiological investigations of patients. Mortality associated with these viruses can be better assessed by calculating the excess mortality that occurs during the periods of the epidemic^14^ ^,^ ^15^. Using a similar methodology, it can be estimated that in New York city, the number of excess deaths during the first 4 weeks of the epidemic was 12,547, while the conventional epidemiological surveillance was only able to identify 7,186 deaths due to COVID-19, which accounted for 57% of the excess deaths during the period^16^. In Portugal, the excess mortality that occurred between March 1 and April 22 was 3 to 5 times higher than the official reported number of COVID-19 deaths^17^. This underestimation of mortality through surveillance systems can occur because of several factors, such as failure to report the cases, difficulty in identifying cases with atypical presentation, and late complications of the disease. Moreover, several associated factors can also lead to underestimation of mortality through surveillance systems e.g., failure to notify the laboratory results of patients due to case definition criteria. In fact, an unknown proportion of patients with COVID-19 develop serious and potentially fatal cardiovascular complications such as acute coronary syndrome, myocarditis, arrhythmia, and cardiogenic shock; these cases might not be identified by the attending physicians in routine situations^18^.

All-cause mortality exceeded the upper limit of the confidence interval (95%) a week before the first laboratory-confirmed death due to COVID-19 in the cities of São Paulo and Rio de Janeiro. This suggests that there might have already been many deaths that were not captured by the conventional epidemiological surveillance system, which records individual cases. Most cases of COVID-19 are characterized by mild symptoms that can be confused with influenza or even respiratory syncytial virus infection that occurs at this time of year in these two Brazilian cities^19^. Therefore, it is plausible that SARS-CoV-2 had spread widely, and this spread was not identified at the beginning of the Brazilian epidemic. Since the condition of patients with COVID-19 usually worsens around the tenth day after the onset of symptoms and death often occurs 3 weeks after the onset of symptoms^20^, community transmission might have occurred before the first case was officially identified. The County of Santa Clara Medical Examiner-Coroner detected SARS-CoV-2 during the autopsy of a patient who died at home from an unknown cause on February 6 and had no history of travel abroad. The first case of community transmission in the USA was identified by regular epidemiological surveillance on February 26^21^. During the 2009 influenza pandemic in the State of São Paulo, it was observed that mortality from respiratory diseases in the population was already above the upper limit of the 95% confidence interval 2 weeks before confirmation of community-level viral transmission in the state^14^. These findings reinforce the sensitivity and usefulness of surveillance of excess deaths and other strategies in complementing traditional epidemiological surveillance based on the notification and investigation of suspected clinical cases. 

Another notable aspect was the fact that excess mortality until EW 16 had a strong Pearson correlation with official mortality (r = 0.94), which reinforces the consistency of the findings. The slope of the regression suggests that the number of excess deaths in the six cities was 2.5 times higher than the number of deaths identified by the official surveillance system at that time. Due to the small number of cases used to build this model, it cannot be assumed that an equal proportion of excess deaths due to COVID-19 will be seen in other cities or that this pattern will be maintained throughout the pandemic in the cities analyzed. 

The present study has some limitations, many of which are attributed to the fact that the assessment was made when the epidemic was still in its initial stages; hence, some data might still be incomplete. In addition, the death records were obtained from the registry information system instead of the Mortality Information System (SIM, acronym in Portuguese), which checks the consistency of the diagnosis and follows the recommendations of the WHO for the classification of the basic cause of death. This was because SIM takes at least 6 months to consolidate the information and provide free access to data for research. However, this limitation is restricted to the analysis of the excess of deaths from respiratory causes since the cause of death does not affect the assessment of mortality from all causes. Another limitation related to the use of notary data is that in this system, the data are available only for the years 2019 and 2020; therefore, we were unable to calculate the average mortality rate over several years, which would have been beneficial^15^ ^,^ ^16^. Despite these limitations, the results were consistent across all age groups severely affected by the pandemic, and there was a strong correlation between the mortality documented in the official surveillance system and excess mortality. Moreover, the period of increase in mortality coincided with the period of the pandemic. No excess deaths were reported in the city with a lower incidence of COVID-19.

Excess mortality is a tool used to assess mortality associated with climatic events, earthquakes, and epidemics with good temporal definition such as those caused by the influenza virus, respiratory syncytial virus, and chikungunya^14^ ^,^ ^15^ ^,^ ^22^. Similar to the CDC in the USA, the EuroMOMO Project tracks the weekly mortality data of some European countries to assess the excess mortality and monitor the impact of influenza epidemics. These systems have been able to capture the excess mortality associated with the ongoing COVID-19 pandemic^16^ ^,^ ^23^. Analyzing the data of excess deaths, regardless of the cause recorded on death certificates, seems to be the best method to estimate of mortality during this pandemic^24^. The phenomenon of excess mortality cannot be exclusively attributed to the SARS-CoV-2, as some proportion of these deaths would have been caused by the excess burden on the health care network. However, these deaths can be considered a part of the pandemic burden as they would not have occurred in its absence.

Surveillance of excess mortality is a simple, low-cost, easy-to-implement tool that can complement traditional epidemiological surveillance. Therefore, its use should be expanded to other countries and be promoted by the WHO. An important characteristic of this approach is that it does not depend on the flow of investigation of cases by healthcare professionals who are involved in preparedness and response activities during major epidemics. Since it does not necessarily depend on laboratory research, surveillance of excess mortality can be useful, especially in low-income countries. Moreover, it does not depend on initial clinical suspicion and can be used to determine the mortality associated with different atypical phenomena, such as epidemics, extreme weather events, or earthquakes.
